# AZD5153, a Bivalent BRD4 Inhibitor, Suppresses Hepatocarcinogenesis by Altering BRD4 Chromosomal Landscape and Modulating the Transcriptome of HCC Cells

**DOI:** 10.3389/fcell.2022.853652

**Published:** 2022-03-24

**Authors:** Cho-Hao Lin, Jimmy Chun-Tien Kuo, Ding Li, Aaron B. Koenig, Alexander Pan, Pearlly Yan, Xue-Feng Bai, Robert J. Lee, Kalpana Ghoshal

**Affiliations:** ^1^ Department of Pathology, College of Medicine, Columbus, OH, United States; ^2^ Comprehensive Cancer Center, The Ohio State University, Columbus, OH, United States; ^3^ Division of Pharmaceutics and Pharmacology, College of Pharmacy, Columbus, OH, United States

**Keywords:** HCC, BRD4 (bromodomain-containing protein 4), AZD5153, NAPRT, ChIP-seq, nanoemulsion

## Abstract

BRD4, a chromatin modifier frequently upregulated in a variety of neoplasms including hepatocellular cancer (HCC), promotes cancer cell growth by activating oncogenes through its interaction with acetylated histone tails of nucleosomes. Here, we determined the anti-HCC efficacy of AZD5153, a potent bivalent BRD4 inhibitor, and elucidated its underlying molecular mechanism of action. AZD5153 treatment inhibited HCC cell proliferation, clonogenic survival and induced apoptosis in HCC cells. *In vivo*, AZD5153-formulated lipid nanoemulsions inhibited both orthotopic and subcutaneous HCCLM3 xenograft growth in NSG mice. Mapping of BRD4- chromosomal targets by ChIP-seq analysis identified the occupancy of BRD4 with the promoters, gene bodies, and super-enhancers of both mRNA and noncoding RNA genes, which were disrupted upon AZD5153 treatment. RNA-seq analysis of polyadenylated RNAs showed several BRD4 target genes involved in DNA replication, cell proliferation, and anti-apoptosis were repressed in AZD5153-treated HCC cells. In addition to known tumor-promoting genes, e.g., c-*MYC*, *YAP1*, *RAD51B*, *TRIB3*, *SLC17A9*, *JADE1*, we found that *NAPRT*, encoding a key enzyme for NAD^+^ biosynthesis from nicotinic acid, was also suppressed in HCC cells by the BRD4 inhibitor. Interestingly, AZD5153 treatment upregulated *NAMPT*, whose product is the rate-limiting enzyme for NAD^+^ synthesis from nicotinamide. This may explain why AZD5153 acted in concert with FK866, a potent NAMPT inhibitor, in reducing HCC cell proliferation and clonogenic survival. In conclusion, our results identified novel targets of BRD4 in the HCCLM3 cell genome and demonstrated anti-HCC efficacy of AZD5153, which was potentiated in combination with an NAMPT inhibitor.

## Introduction

Liver cancer is one of the deadliest cancers globally (Global Burden of Disease Liver Cancer et al., 2017; Llovet et al., 2021a). According to the National Cancer Institute, liver and intrahepatic bile duct cancer had an incidence of 8.9 cases per 100,000 persons living in the United States in 2015, and death rates due to liver cancer increased 2.5% yearly between 2006 and 2015. According to data from the Surveillance, Epidemiology, and End Results (SEER) program, HCC has been the fastest-growing cause of mortality due to cancer in the United States since the early 2,000s and is projected to become a leading cause of cancer death by 2030 without intervention (Rahib et al., 2014). HCC is most commonly a sequel of chronic liver disease caused by chronic viral hepatitis, alcohol-induced injury, non-alcoholic fatty liver disease, exposure to aflatoxin B1, and several metabolic diseases (Singal et al., 2020; Konyn et al., 2021). Treatment modalities include resection, ablation, chemoembolization, or liver transplant (Forner et al., 2019; Yarchoan et al., 2019). Patients with advanced-stage liver cancer at the time of diagnosis or after treatment failure achieve survival benefit from a systemic therapy with multi-kinase inhibitors such as sorafenib or lenvatinib (Kudo, 2020; Finn and Zhu, 2021). Very recently, several systemic targeted therapies such as atezolizumab plus bevacizumab, sorafenib, lenvatinib, regorafenib, cabozantinib and ramucirumab have been approved by the FDA (Llovet et al., 2021a; Llovet et al., 2021b). Amongst these, the combination of atezolizumab, an anti-PD-L1 antibody, and bevacizumab, an anti-VEGF antibody approved as the first-line therapy for advanced HCC, has prolonged HCC patient survival at least 2-fold compared to sorafenib.

BRD4 is a member of the bromodomains and extraterminal domain (BET) family that functions as a transcriptional coactivator (Fujisawa and Filippakopoulos, 2017; Hsu and Blobel, 2017). It functions as a reader of histone post-translational modification by its two bromodomains that cooperatively bind acetylated lysine residues. BRD4 also harbors a C-terminal motif (CTM) that facilitates the recruitment of transcriptional regulators. Lysine methylation or serine/threonine phosphorylation of nucleosomal histones also modulates the affinity of BRD4 for its target loci (Fujisawa and Filippakopoulos, 2017). Studies have shown the importance of enhancers and super-enhancers that are characterized by high occupancy of BRD4 and H3K27ac in aberrantly upregulated oncogenes (Chapuy et al., 2013; Hnisz et al., 2013; Lovén et al., 2013). Several mechanisms are involved in mediating transactivating functions of BRD4. BRD4 facilitates transcription by interacting with the Mediator complex to bring enhancers and promoters into proximity (Lovén et al., 2013; Fujisawa and Filippakopoulos, 2017). It also promotes transcriptional elongation by recruiting the p-TEFb kinase, which then phosphorylates RNA polymerase II (Yang et al., 2005).

NUT midline carcinoma is characterized by oncogenic transformation caused, in most cases, by a BRD-NUT fusion protein that drives chromatin remodeling and *MYC* overexpression (Delmore et al., 2011; Grayson et al., 2014). *BRD4* is upregulated in diverse types of cancer, resulting in the induction of potent oncogenes such as *MYC*, *c-MYB*, *ERG*, and *E2F1* (Fujisawa and Filippakopoulos, 2017; Liang et al., 2021). In HCC, increased *BRD4* expression is associated with higher mortality, which correlates with both MYC-dependent and MYC-independent tumor-promoting activity (Li et al., 2016; Choi et al., 2021).

Pharmacologic inhibition of BRD4 has been widely studied as a potential therapeutic target in cancers (Filippakopoulos et al., 2010; Delmore et al., 2011; Qi, 2014). Several related first-generation, small-molecule BET inhibitors, e.g., JQ1, iBET762, and OTX015, have demonstrated antitumor activity in preclinical models of solid and liquid tumors. These drugs are able to dissociate BET proteins from super-enhancer (SE) regions, causing suppression of *MYC* and *YAP1* expression as well as a reduction in NF-κB activity (Fujisawa and Filippakopoulos, 2017; Zanconato et al., 2018). The very first BRD4 inhibitor, JQ1, has exhibited anti-tumorigenic functions in diverse hematologic malignancies and solid tumors (Delmore et al., 2011; Lockwood et al., 2012; Lovén et al., 2013; Qi, 2014; Li et al., 2016; Kohnken et al., 2018) by selectively repressing oncogenes that are under the transcriptional control of super-enhancers in certain cancer cells. When administered in combination therapy with the HDAC inhibitor SAHA, JQ1 induces expression of the *P57* (*CDKN1C*) tumor suppressor in mouse pancreatic ductal adenocarcinoma, which correlates with increased acetylation of histone H3 tails at the *P57* promoter (Mazur et al., 2015).

AZD5153, a bivalent inhibitor of BET bromodomains developed by AstraZeneca, binds to both bromodomains of BRD4 with triazolopyridazine and N-methylpiperazinone moieties (Rhyasen et al., 2016). AZD5153 exhibited improved potency compared to single bromodomain inhibitors, e.g., JQ1 in inhibiting the growth of diffuse large B-cell lymphoma, acute myelogenous leukemia, and multiple myeloma cells *in vitro* and in mouse hematologic xenografts (Rhyasen et al., 2016). AZD5153 also exhibited anti-tumorigenic functions in prostate, colorectal, and thyroid cancers and in Wilm’s tumor (Shen et al., 2018; Xu et al., 2018; Zhang et al., 2019; Woods et al., 2021). Further, this therapy decreased the blood level of *MYC* RNA and increased that of *HEXIM1* in a human tolerability study (Rhyasen et al., 2016). Recently, a Phase 1 clinical trial of AZD5153 in patients with relapsed or refractory solid tumors including breast, ovarian, pancreatic, prostate cancers and lymphoma has been completed (NCT03205176). However, the therapeutic efficacy of AZD5153 has not been tested in HCC patients or in preclinical models. Analysis of independent patient cohorts revealed that BRD4 is upregulated in HCCs, which correlated with higher mortality (Li et al., 2016; Choi et al., 2021). In this study, we have determined the efficacy of AZD5153 in pre-clinical HCC models and assessed its effect on the transcriptome and on the expression of BRD4 targets in HCC cells. In particular, we implicate NAD^+^ metabolism as an object of BRD4 regulation in HCC.

## Materials and Methods

### Materials

Miglyol 812 (MCT) was purchased from Sasol Germany GmbH (Hamburg, Germany). Egg L-α-phosphatidylcholine (EPC) was purchased from Avanti Polar Lipids (Alabaster, AL, United States). Tween 80, chloroform, and other reagents were purchased from Fisher Scientific (Hampton, NH, , United States). AZD5153 6-Hydroxy-2-naphthoic acid salt (AZD5153) and FK866 were purchased from MedChemExpress (Monmouth Junction, NJ, , United States) and LC Laboratories (Woburn, MA, , United States), respectively.

### HCC Cell Culture and Survival Assay

HCC cell lines (HepG2, Hep3B, PLC/PRF/5, SNU-387, SNU-449) were obtained from ATCC (Manassas, VA, United States). Huh-7 and HCCLM3 cells were provided by Dr. James Taylor (Fox Chase Center, PA, United States) and Dr. Hangxiang Wang (The First Affiliated Hospital, School of Medicine, Zhejiang University, Hangzhou, China), respectively. Cells were maintained in either DMEM or Minimum Essential Media supplemented with l-glutamine (2 mM), 10% FBS, sodium pyruvate (1 mM), and penicillin/streptomycin/amphotericin (Thermo Fisher Scientific) at 37°C with 5% CO_2_.

HCC cells plated in 96-well plates (3,000 cells/well) were cultured overnight followed by the treatment with AZD5153, FK866, a combination of both drugs, and vehicle (DMSO). Cell survival was measured after 72 h of drug treatment using CellTiter-Glo Luminescent Cell Viability Assay (Promega, Madison, WI, United States). Each treatment was done in quadruplicate.

### Clonogenic Survival Assay

HCC cells were seeded in a 12-well plate (5.0 × 10^4^ to 1.0 × 10^5^ cells/well) 24 h before treatment. After 24 h, cells were treated with AZD5153, FK866, a combination of both drugs and vehicle (DMSO) for 5 days. Drug media were changed every other day to maintain active drug concentrations. Media were removed after 5 days and cells were washed with PBS twice, fixed with formalin (phosphate-buffered, 10%, Fisher Scientific) for 30 min, and visualized by 0.01% crystal violet in MilliQ water. The plates were washed thoroughly using MilliQ water to remove the residual dye. Quantification of cell viability was done by dissolving crystal violet solids using 100% methanol. Methanol was added to each well and mixed thoroughly on an orbital shaker. Absorbances at 540 nm were taken by a Molecular Devices Spectramax M5. All data were shown in percentage after normalization to DMSO control.

### Real-Time RT-PCR Analysis

Total RNA was isolated from cells using TRIzol reagent, treated with DNase 1, and converted into cDNA using a high-capacity cDNA reverse transcription kit (Applied Biosystems, Foster City, CA) according to the manufacturer’s instructions. Subsequently, qPCR was performed in a thermocycler using 0.01–0.1 μg cDNA and SYBR Green PCR mix. The fold changes in gene expression normalized to 18S rRNA or β-actin mRNA were calculated using the ΔΔC_T_ method. The forward (F) and reverse (R) primer sequences for qPCR are provided below.

c-MYC-F, TGA​GGA​GAC​ACC​GCC​CAC; c-MYC-R, CAA​CAT​CGA​TTT​CTT​CCT​CAT​CTT​C; DDX54-F, CAA​GTG​GGA​CCG​TAA​GAA​GAA; DDX54-R, CTT​GTA​GGA​GCT​GCT​GAT​GTA​G; GAR1-F, GCA​AAG​TGC​AAC​GGA​ATA​GTG; GAR1-R, TTA​TTG​AGC​ACC​CAC​AGA​GTG; FOXA3-F, TCT​ACT​ACC​AGG​GCC​TCT​ATT​C; FOXA3-R, GAA​GTG​TCA​CCA​GAA​GGA​TCA​G; JADE1-F, TGT​GAG​TGG​GTT​GTG​GAT​ATG; JADE1-R, GAA​GGT​CAA​GTG​GAC​TGG​TAA​A; MCM2-F, AAG​CCA​GGA​GAC​GAG​ATA​GA; MCM2-R, CTA​GGA​TGA​CAG​TGG​CAA​AGA; MCM3-F, GAT​CAC​CAG​ACC​ATC​ACC​ATC; MCM3-R, TCA​CCA​GGC​TTC​GCT​TTA​TC; MCM5-F, CAA​TGA​GGA​GAG​GGA​TGT​GAT​G; MCM5-R, CAC​TCG​GCA​GTA​GGC​AAT​AA; MCM10-F, GGT​TCA​GAG​GAG​GTG​TGT​TTA​T; MCM10-R, CGG​CTC​TCC​ATT​CTT​CTT​CTT; NAPRT-F, TAC​AGC​AGG​CTC​AGG​AGA​T; NAPRT-R, TTG​TTG​CTG​ACT​ACG​ATG​AGG; NFĸB-F, ATC​CCA​TCT​TTG​ACA​ATC​GTG; NFĸB-R, CTG​GTC​CCG​TGA​AAT​ACA​CCT​C; RAD51-F, GGC​AGT​GAT​GTC​CTG​GAT​AAT​G; RAD51-R, CGG​TGG​CAC​TGT​CTA​CAA​TAA​G; RBMX-F, CAC​CAC​CAC​CAC​GAG​ATT​ATA​C; RBMX-R, GTC​ACG​ATC​ACG​ACC​ATA​TCC; SMARCAL1-F, CAG​GGT​TCA​AGG​GTT​CTC​ATT; SMARCAL1-R, TTG​TCC​ATC​CAG​TCG​ACA​ATA​C; TOPBP1-F, TGG​ACG​AAG​TAG​AGT​CCT​AGA​G; TOPBP1-R, GCT​GTA​GGG​TCA​TCC​CAA​AT; TRIB3-F, CAA​GGT​TGA​GGG​ACA​GGA​TTA​G; TRIB3-R, TAG​AGT​ATG​GAC​CTG​GGA​TTG​T; USF2-F, CGG​AGG​GAC​AAG​ATC​AAC​AA; USF2-R, TGG​ACA​GGA​TCC​CTC​CTT​TA; YAP1-F, AAA​CAG​CAA​GAA​CTG​CTT​CG; YAP1-R, GCC​AAA​ACA​GAC​TCC​ATG​TC; 18S rRNA-F, GTA​ACC​CGT​TGA​ACC​CCA​TT; 18S rRNA-R, CCA​TCC​AAT​CGG​TAG​TAG​CG; β-actin-F, CTG​GCA​CCA​CAC​CTT​CTA​CAA​TG; β-actin-R, TAG​CAC​AGC​CTG​GAT​AGC​AAC​G.

### Western Blot Analysis

HCC cells (1 × 10^6^ cells) were seeded overnight in a 100 mm dish and treated with AZD5153 or DMSO for 24 h. Whole-cell lysates were prepared in cell lysis buffer (#9803, Cell signaling technology (CST), Beverly, MA, United States) supplemented with protease inhibitor cocktails (Sigma, St. Louis, MO, United States). The cell lysates were incubated at 4°C for 10 min and centrifuged at full speed for 10 min to collect supernatants. Equivalent amounts of protein lysates were separated in SDS-polyacrylamide 4–15% gradient gels (Bio-Rad, Hercules, CA, United States), transferred to nitrocellulose membranes (GE Healthcare, Chicago, IL, United States), pre-incubated in blocking buffer (3% BSA in Tris-buffered saline with 0.1*%* Tween 20) followed by immunoblotting with BRD4 (#13440, CST)-, c-MYC (#13987, CST)-, NAPRT (#PA5-100073, Invitrogen), YAP1 (#14074, CST)-or β-actin- (#66009-1-Ig, Protein Tech) specific antibodies. After incubation with appropriate secondary antibody conjugated to IRD-680 or IRD-800 (LI-COR, Lincoln, NE, United States), the immune-reactive bands were detected using Odyssey CLx Imaging System (LI-COR) and quantified using Image Studio software (LICOR).

### Estimation of Intracellular NAD^+^ and NADH Levels

HCC cells (5,000 cells/well) were seeded in 96-well plates and allowed to grow overnight at 37°C followed by the treatment with AZD5153, FK866, a combination of both drugs, and vehicle (DMSO). The overall NAD^+^/NADH level in the cells was measured after 48 h of drug treatment using NAD/NADH-Glo Assay (Promega). The overall NAD^+^/NADH level was normalized to cell counts determined by crystal violet staining as stated above. Quantification of the cell counts was done by dissolving crystal violet in DMSO. Data are shown in percentages after normalization to the value of the DMSO control. Each treatment was done in triplicate.

### Cell Cycle and Apoptosis Assay by Flow Cytometry

Cells at distinct phases of the cell cycle were analyzed by flow cytometry after staining DNA with propidium iodide (PI). HCC cells treated with AZD5153 or DMSO for 24 h were harvested by trypsinization, washed with phosphate-buffered saline (PBS), and fixed in ice-cold 70% ethanol for 60 min. The cells were centrifuged, washed with PBS, resuspended in PBS (0.5 ml), and treated with 50 μL of RNase A (1 mg/ml in PBS) followed by the addition of 50 μL of propidium iodide (PI) (500 μg/ml in PBS) with gentle mixing. The mixture was incubated in the dark at room temperature for 15 min and subjected to flow cytometry using an LSRII flow cytometer (BD Biosciences, CA, United States). Apoptotic cells were quantified by flow cytometry after staining cells with PI and Annexin V using Annexin V/Dead Cell Apoptosis kit (Thermo Fisher Scientific) following the manufacturer’s protocol.

### Preparation of AZD5153 in Lipid Nanoemulsion (AZD-NE)

Lipid nanoemulsion of AZD5153 (AZD-NE) was prepared by the chloroform emulsion-solvent evaporation method followed by homogenization. The lipid composition used was Miglyol 812/EPC/Tween 80 at a weight ratio of 57:15:25. Briefly, lipids were dissolved in chloroform and mixed with AZD5153 dissolved in DMSO. The lipid-drug mixture was then rapidly injected into phosphate buffer (PB, 20mM, pH 7.4) with a syringe and an 18G needle. The lipid nanoemulsion was then mixed uniformly by vortexing for 30 s. After injection, high-pressure homogenization was applied to break down the particle sizes by an Avestin-Emulsiflex-C5 homogenizer. After homogenization, chloroform was removed through evaporation by a rotary evaporator inside a 60°C water bath. Sucrose was added afterward as a cryoprotectant (10%, w/v). The nanoemulsion solution was sterilized by filtration through 0.45 μm PES syringe filters and frozen at−20°C until use.

The particle size and zeta potential of AZD-NE were determined by dynamic light scattering with a NICOMP Z3000 Nano DLS/ZLS System (Entegris, Billerica, MA, United States). The drug concentrations of AZD-NE were determined by reverse-phase high-performance liquid chromatography (HPLC). The analysis was run with an isocratic 80% methanol/20% water mobile phase run at 1 ml/min on a C18 column (Kromasil 100-5-C18, 4.6 × 150 mm). The photodiode-array (PDA) detector was set at 303 nm for AZD5153 detection.

### Chromatin-Immunoprecipitation-Sequencing Analysis

HCCLM3 cells were treated with 10 μM of AZD5153 or DMSO for 24 h and processed for ChIP assay using an Active Motif kit (Active Motif, Carlsbad, CA, United States) following the kit instructions. Two biological replicates were used for this experiment. ChIP-seq was performed by Active Motif using FactorPath ChIP-Seq technology (https://www.activemotif.com/catalog/1072/factorpath). HCCLM3 cells (AZD5153- or DMSO- treated) in culture media containing 1% formaldehyde were incubated at 37°C for 15 min followed by quenching with 0.125 M glycine. The chromatin was isolated in the lysis buffer supplied in the kit and the DNA was sheared by sonication to ∼300–500 bp. An aliquot of chromatin (25 μg) was precleared with protein A agarose beads followed by immunoprecipitation with anti-BRD4 antibody (4 μg) (#A301-985A1 Bethyl Labs) or anti-histone H3K27Ac antibody (#39135, Active Motif). Immune complexes pulled down with protein A agarose beads were washed, eluted from the beads with SDS buffer, and digested successively with RNase A and proteinase K. Crosslinks were reversed by incubation overnight at 65°C. ChIP-DNA and input DNA (without immunoprecipitation) were purified by phenol-chloroform extraction and ethanol precipitation.

ChIP-seq bioinformatic analysis was performed by using FactorPath ChIP-Seq technology of Active Motif. To this end, Illumina sequencing libraries were prepared from the ChIP and input DNAs by successive enzymatic end-polishing, dA-addition, and adaptor ligation. After PCR amplification, the DNA libraries were quantified and sequenced on Illumina’s NextSeq 500 (75 nucleotide reads, single end). Reads were aligned to the human genome (hg38) using the BWA algorithm. Only uniquely mapped reads (mapping quality >= 25) after removal of duplicate reads were used for subsequent analysis. Alignments were extended *in silico* at the 3′-ends to a length of 200 bp, the average genomic fragment length in the size-selected library and assigned to 32-nt bins along the genome. The resulting histograms (genomic “signal maps”) were stored in bigWig files. Peak locations were determined using the MACS algorithm (v1.4.2) with a cutoff of *p*-value = 1 e-7. Signal maps and peak locations were used as input data to the Active Motifs proprietary analysis program, which creates Excel tables containing detailed information on sample comparison, peak metrics, peak locations, and gene annotations. Average Plots and Heatmaps were generated with the R Bioconductor package “seqplots”. For gene ontology (GO) analysis, GREAT functional annotation tool was utilized. The data is presented as a table of pathways significantly affected by BRD4 binding. *p* values are presented in–log_10_. The raw sequence data has been submitted to Geo database (accession # GSE172186).

### RNA Sequencing Analysis

RNA-seq was performed in HCCLM3 cells treated with AZD5153 (10 μM) or DMSO for 24 h. Two biological replicates were used for this experiment. Total RNAs isolated with TRIzol reagent were quantified using Qubit Fluorometer, and those with RIN values > 7 were used for polyA + RNA isolation using NEBNext Poly (A) mRNA Magnetic Isolation Module (#E7490, New England Biolabs, NY). Purified mRNAs were fragmented and cDNAs were generated using NEBNext^®^ Ultra™ II Directional (stranded) RNA Library Prep Kit for Illumina (NEB #E7760L) and NEBNext Multiplex Oligos for Illumina Unique Dual Index Primer Pairs (NEB #6442S/L) Libraries were amplified 12X and sequenced with Illumina NovaSeq 6,000 flow cell using paired-end 150-bp format to at least 17 million pass-filter clusters per sample (equivalent to 34 million reads). The sequencing data was submitted to Geo database (Accession # GSE181406).

### RNA-Seq Data Analysis

Bulk mRNA-seq data were analyzed as follows: First, raw FASTQ reads were quality- and adapter-trimmed using AdapterRemoval (v2.2.0) (Kroll et al., 2014). HISAT2 (v2.0.6) (Kim et al., 2019) was then used to align trimmed reads to a combined reference of NCBI RefSeq human mtDNA, human rRNA, and PhiX bacteriophage sequences to remove unwanted reads. Filtered reads were then aligned to the GRCh38 genome using HISAT2. Assessment of post-alignment quality control metrics (e.g., alignment, exonic, and duplication rates) was performed using QuaCRS (Kroll et al., 2014). GENCODE (V25) annotated gene counts were quantified using Subread featureCounts (v1.5.1) (Liao et al., 2014).

Standard DESeq2 (v1.20.0) (Love et al., 2014) analysis was used to identify differentially expressed genes (DEG) across treatment and cell line groups. The DESeq function was used with default parameters to calculate size-normalized expression counts and then estimate log_2_ fold-changes and *p*-values for each gene. Genes were considered differentially expressed (DE) if the Benjamini-Hochberg adjusted *p*-values were ≤0.05 and absolute fold change ≥2.

### Mouse Strains, Animal Husbandry and Treatment

NSG (NOD scid IL2Rγ^−/-^) mice purchased from The Ohio State University Comprehensive Cancer Center Target Validation Shared Resources (TVSR) were housed in a temperature-controlled pathogen-free room under a 12 h light/12 h dark cycle and fed a normal chow diet. All animal studies were reviewed and approved by The Ohio State University Institutional Laboratory Animal Care and Use Committee. Both male and female mice were used for experiments.

For subcutaneous xenografts, 10–12 weeks old NSG mice were injected subcutaneously with HCCLM3 (2.5 × 10^6^) cells into the right flank. Tumor volumes were measured with a digital caliper using the formula (1/2 (length x width^2^). When tumor volume reached 100 mm^3^, mice were randomized into two groups and injected intraperitoneally with AZD-NE (3 mg/kg/day) or empty particles. After 21 days of treatment, mice were euthanized and tumors were harvested, weighed, and photographed. For the orthotopic model, 10–12 weeks old female NSG mice were injected with ∼1 × 10^6^ million HCCLM3-Luc (firefly luciferase-expressing) cells into the left liver lobe, and tumor growth was monitored weekly by IVIS Lumina (Caliper Life Sciences, Waltham, MA) as described (Lin et al., 2019). Once the luciferase signal was detected, mice were randomized into two groups and treated with the vehicle or encapsulated AZD-NE (3 mg/kg/day). After treatment for 3 weeks, mice were euthanized and tumors were harvested, photographed, and weights were recorded. Formalin-fixed tumor tissues were subjected to immunohistochemical analysis with anti-Ki-67 (#9027, CST) antibody.

### Analyses of TCGA LIHC Data

Normalized (log_2_ (normalized count + 1)) RNA-seq data from livers of Liver and Hepatocellular Carcinoma (LIHC) patients and matched clinical data (overall survival and sample type) assembled by The Cancer Genome Atlas (cancergenome.nih.gov) were downloaded from UCSC Xena data browser (xena.ucsc.edu).

### Statistical Analysis

Statistical analyses were performed with GraphPad PRISM software version 5 (GraphPad Software, San Diego, CA, United States). *p* values < = 0.05 were considered significant.

## Results

### AZD5153 Inhibits HCC Cell Growth, Clonogenic Survival, and Induces Cell Apoptosis

**FIGURE 1:**
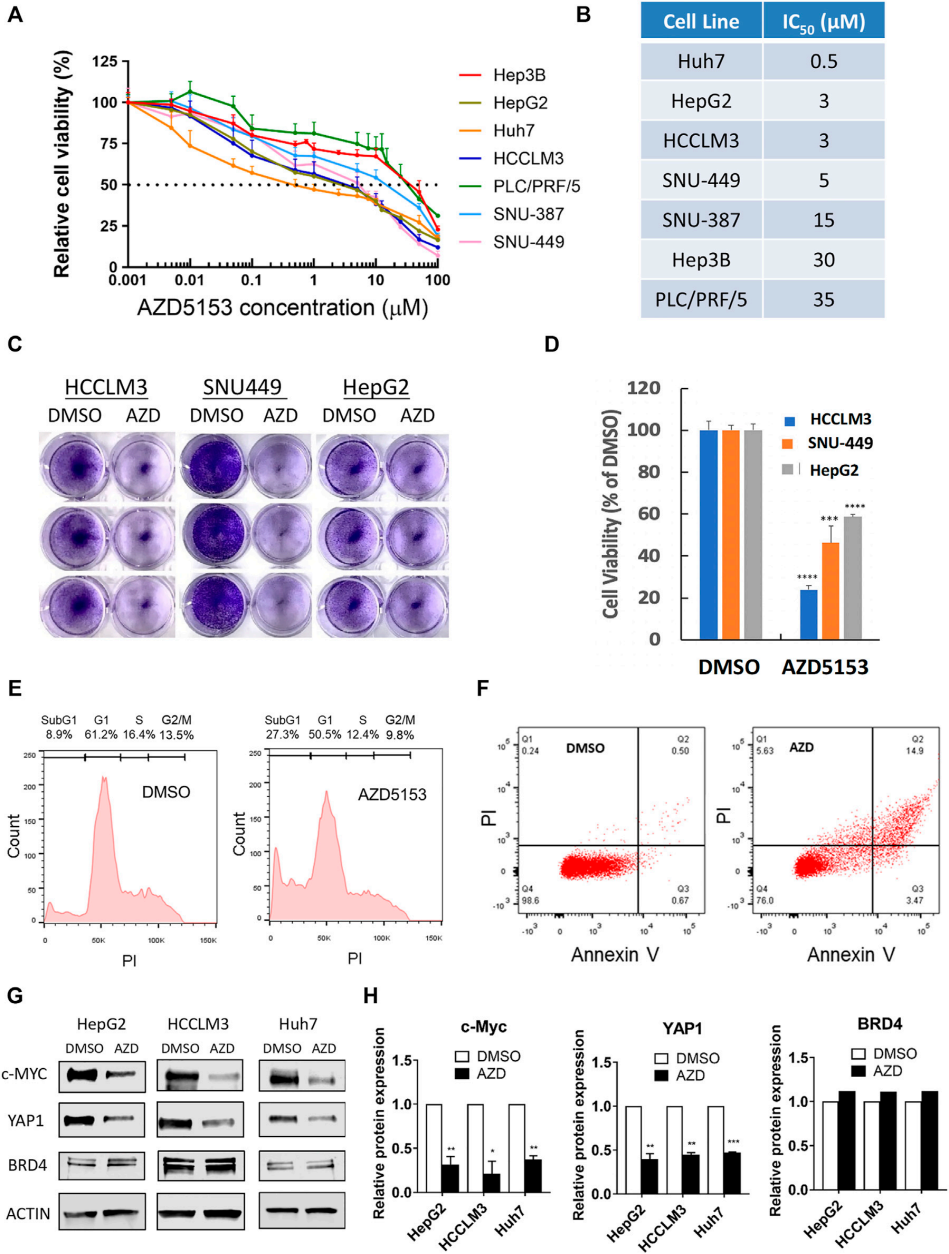
AZD5153 inhibits HCC cell growth and survival and *MYC* and *YAP1* expression. **(A)** HCC cell lines were incubated with AZD5153 at different concentrations ranging from 1 to 100 μM for 72 h, and cell viability was measured using CellTiter-Glo kit, which measures cellular ATP levels (*n* = 4/each drug concentration/cell line). **(B)** AZD5153 IC_50_ values in HCC cell lines derived from data in **(A)**. Data are expressed as mean ± SD. Relative viability was determined relative to that of vehicle (DMSO) treated cells. **(C,D)** Colony formation by HCC cell lines following treatment with AZD5153 (10 μM) or DMSO for 5 days. **(E,F)** HCCLM3 cells treated with AZD5153 (10 μM) for 24 h were subjected to flow cytometry after staining with propidium iodide (PI) **(E)** and Annexin V/PI **(F)**. **(G,H)** Whole-cell extracts of HCC cell lines treated with AZD5153 (10 μM) for 24 h were subjected to immunoblot analysis with c-MYC-, YAP1-, BRD4-, and β-Actin -specific antibodies **(G)**, and the relative c-MYC, YAP1, and BRD4 protein levels were quantified **(H)**. *p* values were determined by Student T-TEST. *, **, *** and **** denote *p*-values < 0.05, 0.01, 0.001, and 0.0001, respectively.

To evaluate the anti-hepatocarcinogenic functions of AZD5153, we determined its IC_50_ in 7 HCC cell lines. AZD5153 reduced cell proliferation dose-dependently in all HCC cell lines tested (Figure 1A). The sensitivity to the drug varied between cell lines, e.g., Huh7 and PLC/PRF/5 cells exhibited the lowest and highest IC_50_ values, respectively, for AZD5153, whereas HepG2, SNU-449, SNU-387, and Hep3B cells showed intermediate IC_50_ values (Figure 1B). AZD5153 also inhibited clonogenic survival of 3 HCC cell lines tested (Figures 1C,D).

Fluorescence-activated cell sorting (FACS) analysis of propidium iodide (PI)-stained HCCLM3 cells showed ∼3-fold increase (8.9–27.3%) in the sub-G1 population when treated with AZD5153 (10 μM) for 24 h compared to the vehicle (DMSO) control indicating that the drug induced apoptosis (Figure 1E). Annexin V-PI staining also showed ∼5-fold increase in the apoptotic cell population (0.67–3.47%) (Figure 1F). These results indicated that AZD5153 inhibited HCC cell growth by promoting apoptosis.

To elucidate the mechanism underlying anti-HCC efficacy of AZD5153, we first assessed the levels of some oncoproteins in HCC cells after drug treatment. It has been reported that BRD4 upregulates the expression of oncogenes such as *c-MYC* and *YAP1*, which were inhibited by treatment with a BRD4 inhibitor such as JQ1. We therefore measured c-MYC and YAP1 levels in AZD5153-treated cells. Immunoblotting confirmed the profound decrease in both proteins after 24 h of AZD5153 treatment in HCCLM3, HepG2, and Huh7 cells, without significant changes in BRD4 level (Figures 1G,H). These results suggested that AZD5153 inhibited BRD4 activity in HCC cells.

### AZD5153-Lipid Nanoemulsion Inhibited HCCLM3 Xenograft Growth in NSG Mice

**FIGURE 2:**
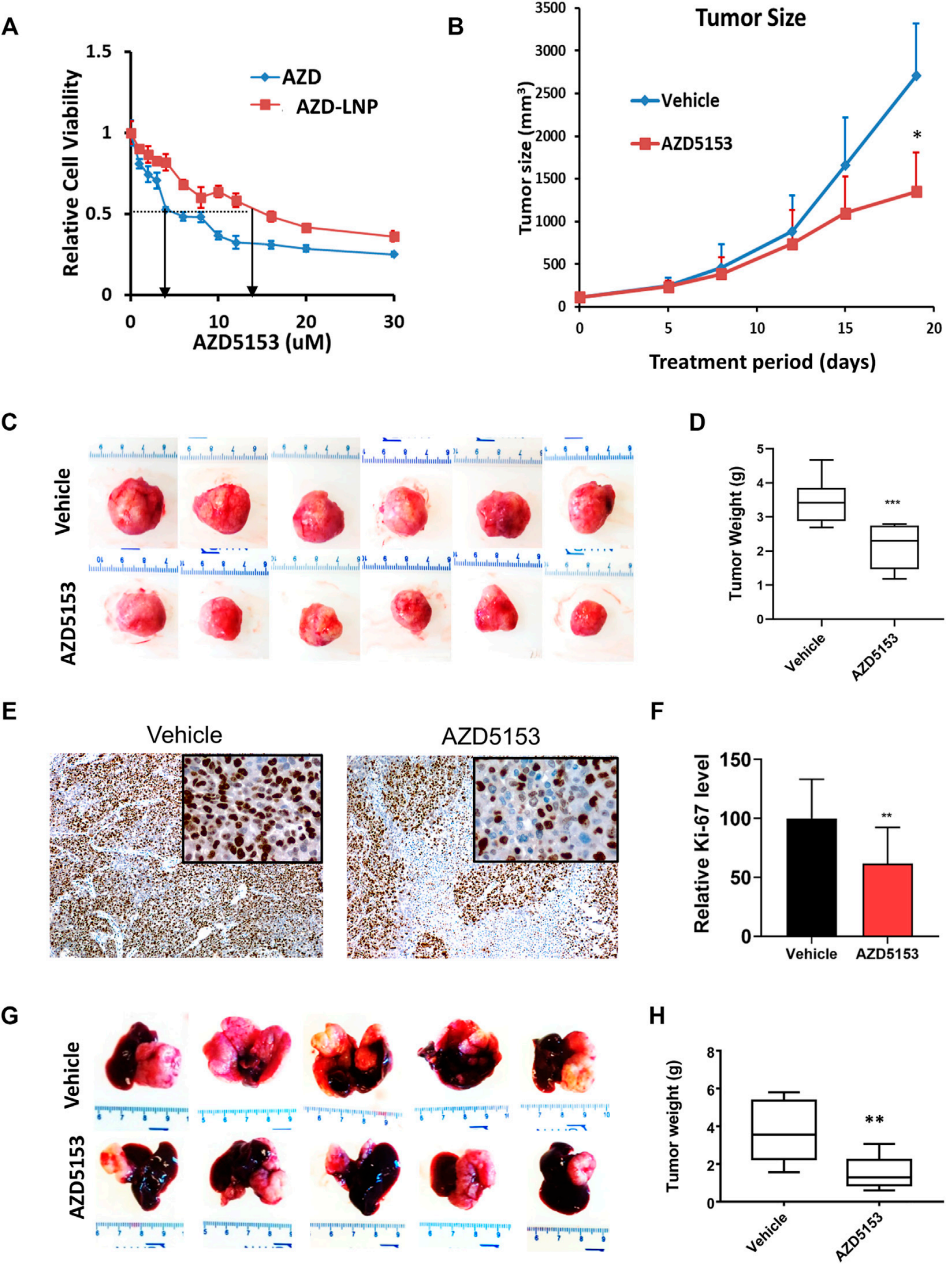
Lipid nanoemulsion (NE)-encapsulated AZD5153 (AZD-NE) inhibits HCC xenograft growth in NSG mice. **(A)** The IC_50_ of AZD-NE and free AZD5153 (dissolved in DMSO) were determined in HCCLM3 using CellTiter-Glo assay. **(B)** The profile of tumor volumes during drug treatment for 18 days of NSG mice bearing subcutaneous HCC xenografts. Mice were injected subcutaneously with 2.5 × 10^6^ HCCLM3 cells into their right flanks. When the tumors reached ∼150 mm^3^, mice were administrated AZD-NE (3 mg/kg/daily) or empty NE alone daily via intraperitoneal (I.P.) injection, and tumor volumes were measured using calipers. **(C,D)** The images **(C)** and average weights **(D)** of subcutaneous tumors were collected from 6 mice after 18 days of drug treatment. **(E,F)** Representative photographs of Ki-67-immunostained tumor sections (Magnification: ×100 and 400X (inset) **(E)** and the quantification of Ki-67 positive tumor cells (N = 4) **(F)**. **(G,H)** Representative liver images and the average tumor weights of the orthotopic liver tumors in NSG mice. Mice were injected orthotopically with 10^6^ HCCLM3-Luc (firefly luciferase expressing) cells into the left liver lobe and treated with AZD-NE (3 mg/kg/daily) or empty NE for 21 days. *p* values were determined by Student T-TEST. *, ** and *** denote *p* values <= 0.05, 0.01, and 0.001, respectively.

To investigate the anti-HCC efficacy of AZD5153 *in vivo*, we first generated the AZD5153 lipid nanoemulsion (AZD-NE) to improve stability and slow release of the drug *in vivo*. To this end, we first characterized the physicochemical properties of AZD-NE. HPLC analysis revealed that AZD5153 concentration within the nanoemulsion was 0.91 mg/ml. The z-average particle size and mean zeta potential of AZD-NE were 92.2 nm (with a polydispersity index [PdI] around 0.227) and −5.07 mV, respectively. In the storage stability study, AZD-NE was found to be stable at 4°C without forming aggregates for at least 28 days (Supplementary Figure S1A). AZD-NE showed higher IC_50_ compared to AZD5153 in HCCLM3 cells (Figure 2A).

To evaluate the efficacy of AZD-NE in suppressing tumor growth *in vivo*, we selected HCCLM3 cells because these cells form tumors within 1–2 weeks when implanted subcutaneously in NSG mice. We started treating mice with AZD-NE (3 mg/kg/day) or the vehicle (empty NE) when the tumor reached ∼100 mm^3^ in size. Mice exhibited body weight loss due to the tumor growth; however, there was no significant difference between the vehicle- and AZD-NE-treated group (Supplementary Figure S1B). After 3 weeks of treatment, tumor weight and volume were significantly reduced by AZD-NE treatment (Figures 2B–D). A significant decrease in the number of Ki-67-positive tumor cells confirmed reduced proliferation of tumor cells upon AZD-NE treatment (Figures 2E,F).

We also tested the therapeutic efficacy of AZD5153 in NSG mice bearing orthotopic (intrahepatic) tumors developed by transplanting HCCLM3 cells expressing firefly luciferase (HCCLM3-Luc). Mice bearing orthotopic xenografts were monitored by bioluminescence imaging (BLI) and were treated with AZD-NE (3 mg/kg/day) or empty NE when the tumor started to grow. At this dose, the body weights of mice of the two groups were comparable (Supplementary Figure S1C). After 3 weeks of treatment, livers with orthotopic tumors were harvested, photographed, tumors were dissected out, and their weights were measured. The results showed that AZD-NE treatment significantly reduced HCCLM3 orthotopic tumor growth (Figures 2G,H).

### Genome-Wide Occupancy of BRD4 in HCCLM3 Cells is Disrupted by AZD5153 Treatment

**FIGURE 3:**
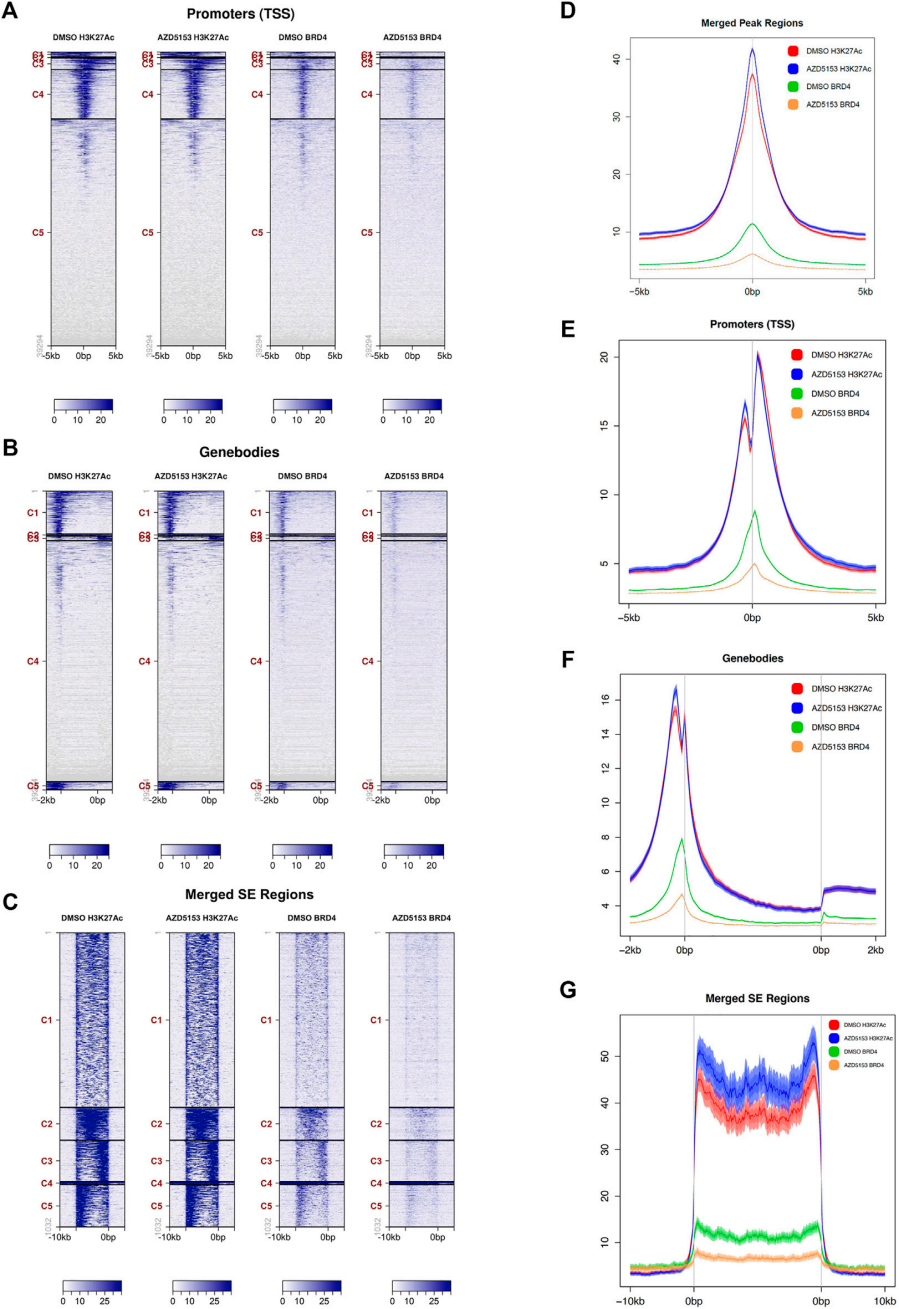
BRD4 occupancy at different chromatin regions of HCCLM3 cells is perturbed after AZD5153 exposure. **(A–C)** ChIP analysis of BRD4 and H3K27Ac occupancy at the promoters, gene bodies and super-enhancer (SEs) in HCCLM3 cells treated with DMSO or AZD55153 (10 μM) for 24 h **(D–G)** Average signal intensity plot of BRD4 and H3K27Ac binding to all peaks, promoters, gene bodies and SEs in the genome of control and AZD5153-treated HCCLM3 cells. The 0bps denote transcription start sites (TSSs) and the regions upstream and downstream of TSS are denoted by−and +, respectively.

BRD4 inhibitors are known to suppress gene expression by dissociating BRD4 from active chromatin marks, especially histone H3K27Ac. We, therefore, sought to identify the chromatin landscape of BRD4 in HCCLM3 cells and to determine whether AZD5153 treatment changes it. To this end, we performed ChIP-seq analysis of formaldehyde-crosslinked chromatin prepared from AZDD5153- and DMSO-treated (control) cells with antibodies specific for BRD4 or histone H3K27Ac, a prototype marker of active chromatin that is highly enriched in super-enhancers (SEs). SEs are defined as gene regulatory elements, usually spanning several thousand bases, that drive expression of genes which play a key role in establishing cell identity (Hnisz et al., 2013). The binding of BRD4 to SEs is known to highly upregulate the expression of certain oncogenes like *MYC* in cancer cells and disruption of this interaction has shown to selectively repress such genes (Chapuy et al., 2013; Hnisz et al., 2013; Lovén et al., 2013). Our ChIP-seq data showed that BRD4 was bound to the promoters, gene bodies, and SEs of control HCCLM3 cells (binding sites of both BRD4 and H3K27Ac in the long arm of chromosome 1 are shown in Supplementary Figure S2A). Occupancy of BRD4 correlated with that of H3K27Ac in the promoter, the gene bodies, and the SEs, although binding of the latter by H3K27Ac was much more robust than that of BRD4 (Figures 3A–C). After AZD5153 treatment, BRD4 occupancy was dramatically reduced, whereas H3K27Ac localization to these genomic regions remained unaffected (Figures 3A–C). Plotting of average signal intensity also showed that BRD4 binding to all three genomic regions dramatically decreased upon AZD5153 treatment, whereas H3K27Ac association to these regions remained largely unaltered (Figures 3D–G). The correlation coefficient (R) is 0.841 between DMSO-BRD4 and DMSO-H3K27Ac, 0.846 between DMSO-BRD4 and AZD5153-BRD4, 0.964 between DMSO-H3K27Ac and AZD5153-H3K27Ac, 0.772 between AZD5153-BRD4 and AZD5153-H3K27Ac, and 0.742 between DMSO-H3K27Ac and AZD5153-BRD4 (Supplementary Figure S2B).

Analysis of genomic loci occupied by H3K27Ac and BRD4 revealed that ∼93% of all chromatin bound BRD4 resided in regions enriched in H3K27Ac marks, suggesting that BRD4 was predominantly recruited to active enhancers in the control HCCLM3 cells (Supplementary Tables S1, S2). BRD4 occupancy was observed in all regions of the genome, but the majority of binding sites were identified in the gene bodies, whereas the lowest number of sites were detected in the proximal downstream regions (Supplementary Table S2). Comparable numbers of BRD4-bound loci were identified in the promoters, introns, and 5′-UTRs. After AZD5153 treatment, BRD4 occupancy was reduced by 70–90% at most of these loci.

**FIGURE 4:**
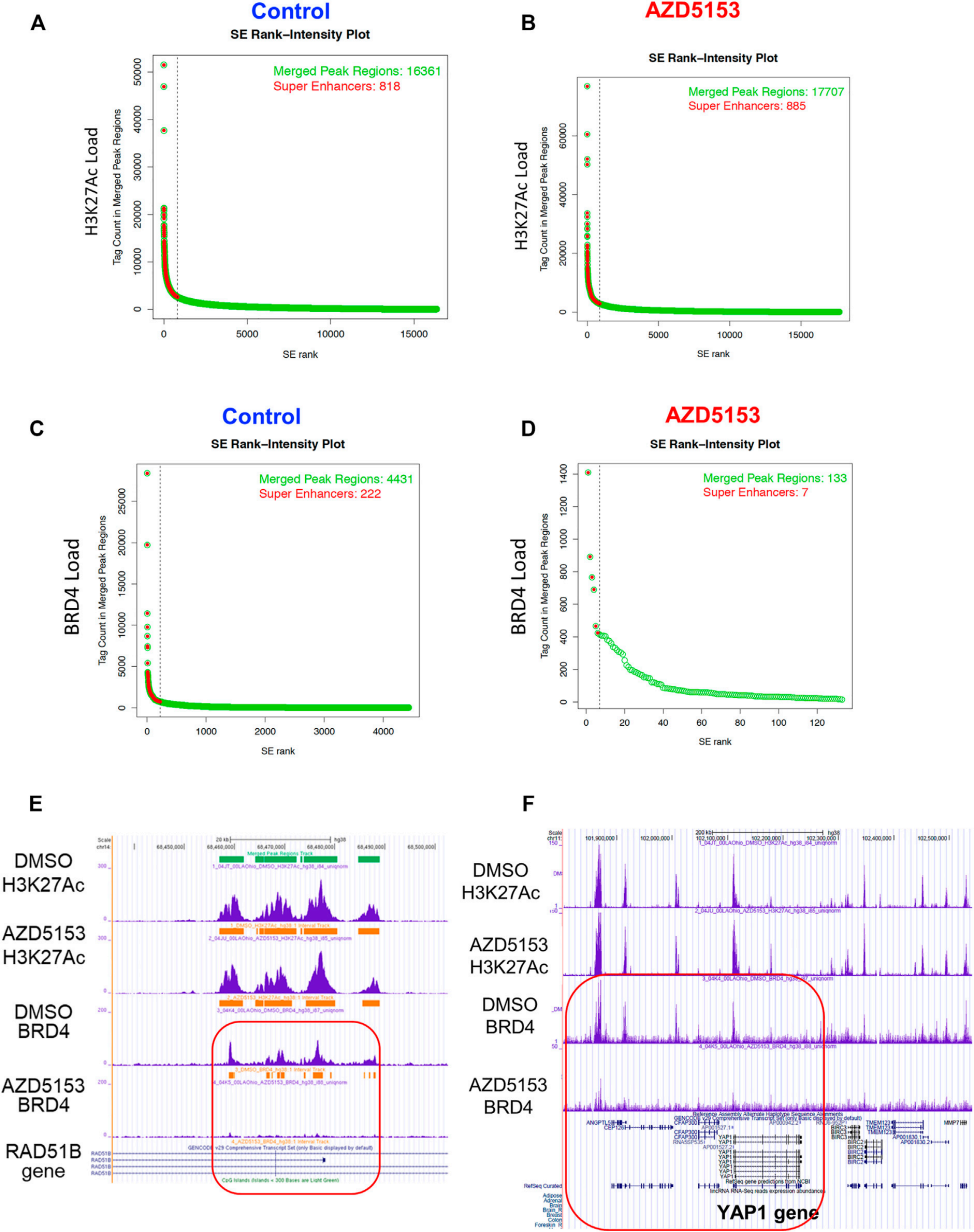
Altered genomic occupancy of BRD4 at super-enhancers (SEs) of HCCLM3 cells after treatment with BRD4 inhibitor. **(A–D)** Hockey stick plot for BRD4 binding across enhancers of HCCLM3 cells following treatment generated from the normalized genome-wide average of reads at the gene. The y axis indicates the average signal (rpm/bp). **(E)** Gene tracks depicting genomic occupancy of BRD4 and H3K27Ac at SEs spanning *VMP1*, *miR21*, *RNU450P*, and *TUBD1* genes in the control and AZD5153-treated HCCLM3 cells. The x and y axes represent the genomic position and the ChIP-seq signal, respectively. Orange horizontal bars denote SEs in this genomic locus. Green and horizontal bars denote localizations of CpG islands and SEs in the genomic region. **(F)** Gene tracks depicting occupancy of BRD4 and H3K27Ac in the genomic region spanning several genes, including *YAP1* in the control and AZD5153-treated HCCLM3 cells. Of note, no SE was identified in this genomic locus.

As presented in the Hockey-stick plots, BRD4 bound to 222 SEs in all chromosomes except chromosomes 13 and 21 in control cells (Figures 4A–D and Supplementary Table S1). AZD5153 treatment profoundly disrupted this association, as BRD4 occupancy remained at only 7 SEs after treatment. In contrast, the association of H3K27Ac was much more robust (818 SEs), which remained comparable (876 loci) after drug exposure. Notably, chromosomes 13 and 21 were also refractory to H3K27Ac binding (Supplementary Table S1). Several SEs located within an intron of *RAD51B* gene were occupied by both H3K27Ac and BRD4 in the control HCCLM3 cells (Figure 4E). As expected, BRD4 association was largely reduced in the AZD5153-treated cells. In contrast, binding of BRD4 and H3K27Ac across the *YAP1* gene was observed in the control HCCLM3 cells; however, no SE was identified (Figure 4F). Here again, only BRD4 binding decreased after AZD5153 treatment.

ChIP-seq data also revealed that the majority of the BRD4-occupied loci in HCCLM3 cells are noncoding RNAs such as long intergenic non-coding RNAs (LINCRNAs), anti-sense RNAs, and microRNAs (Supplementary Table S3). Among these, LINC00824 (*PVT1*), which is adjacent to *c-MYC* gene and frequently upregulated in many cancers, was identified as a BRD4 target. A large number of histone genes including *HIST1H1B*, *HIST1H2BE*, *HIST1H3D*, and *HIST1H4D* were identified as the protein-coding targets of BRD4 (Supplementary Table S3). Additionally, BRD4 was found to be enriched at SEs surrounding (upstream, downstream, or within) *AXL, EHF*, *FOXM1*, *IGF1R*, *RAD51B*, *RAD51D*, *STAT6*, and *TRIB3* genes, most of which are linked to HCC pathogenesis (Adamek and Kasprzak, 2018; Shen et al., 2019; Qi et al., 2020; Batur et al., 2021; Luan et al., 2021). The extent of BRD4 occupancy at these SEs were largely diminished after AZD5153 treatment. These ChIP-seq results identified chromosomal loci, including super-enhancers, occupied by BRD4 in an aggressive HCC cell line and perturbation of this landscape upon exposure to AZD5153, a clinically relevant BRD4 inhibitor.

### AZD5153 Treatment Predominantly Alters the Expression of Genes that Regulate Cell Proliferation, Cell Cycle, and Metabolism in HCCLM3 Cells

**FIGURE 5:**
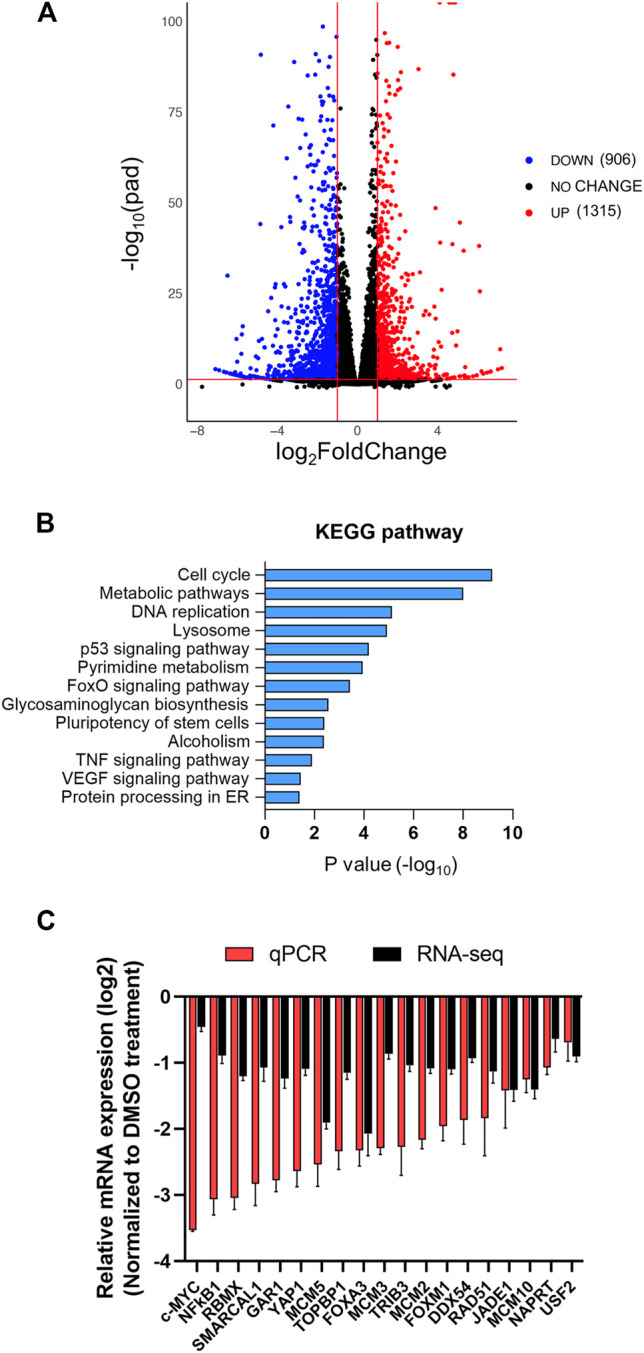
The transcriptome of HCCLM3 cells is altered after AZD5153 treatment. PolyA + RNAs from HCCLM3 cells treated with AZD5153 (10 μM) or DMSO for 24 h were subjected to RNA-seq analysis (see Methods for details). **(A)** A volcano plot depicting the number of differentially expressed genes (FDR-corrected *p*-values (log_10_pad) < 0.05 and log_2_fold-change >= 1 or <= −1). **(B)** KEGG pathway analysis of differentially expressed genes in AZD5153-treated HCCLM3 cells. **(C)** Plot of RNA-seq and RT-qPCR data (log_2_fold-change) of several critical genes downregulated by AZD5153 treatment in HCCLM3 cells.

To gain mechanistic insight on anti-HCC efficacy of AZD5153, we next queried its effect on gene expression of HCC cells. To this end, we performed sequencing of polyadenylated RNAs of HCCLM3 cells treated with AZD5153 (10 μM) or DMSO for 24 h. RNA-seq data demonstrated that 3,412 genes were upregulated and 3,862 genes were downregulated compared to the vehicle control (adjusted *p* values <0.05) in AZD5153-treated cells (Supplementary Table S4). Among these, 906 genes were upregulated and 1,315 genes were downregulated at least 2-fold upon AZD5153 exposure (Figure 5A, Supplementary Table S4). KEGG (Kyoto Encyclopedia of Genes and Genomes) Pathway analysis revealed differential expression of genes that regulate cell proliferation, cell cycle, and metabolism in HCCLM3 cells upon AZD5153 treatment (Figure 5B). Notably, many histone genes, predominantly H2 variants e.g., *HIST2AC/E/I/G*; *HIST2BC*,*D,E*,*F*,*G*,*J*,*K*,*N; HIST2H2AA3*,*AA4; HIST2H2BC*,*D*,*E*,*F; HIST2H3A*,*C*,*D*; and *HIST2H4A*,*B* were highly upregulated (4 to 67-fold) in AZD5153-treated cells (Supplementary Table S4). Notably, cell cycle inhibitors, e.g., *CDKN1A* and *CDKN2D*, were also upregulated in drug treated HCCLM3 cells.

Downregulated genes include well-known oncogenes e.g., *c-MYC* and *YAP1*; several cell cycle genes such as *AURKA*; *BUB1B*; *CCNA2*; *CDC25A*; *CENPL*,*Q*; *E2F* family members (*E2F1*,*2*,*5*,*8*); and MCM (minichromosome maintenance) family members (*MCM2*,*5*,*7*,*10*) in AZD5153-treated cells (Supplementary Table S4). Suppression of these genes correlated with the inhibition of cell survival (Figures 1A,B). Oncofetal genes *AFP* and *H19* were profoundly downregulated in AZD5153-treated cells (Supplementary Table S4). We confirmed the downregulation of several critical genes, including *c-MYC*; *DDX54*; *FOXA3*; *FOXM1*; *JADE1*; *MCM2*,*3*,*5*,*10*; *NFκB1*; *RAD51*; *RBMX*; *TRIB3*; *USF2*; *SMARCAL1*; *TOPBP1*; and *YAP1* by RT-qPCR (Figure 5C). Among these genes, BRD4 SEs were identified spanning *TRIB3*, *DDX54*, and *FOXM1* loci, suggesting their potential direct regulation by BRD4. ChIP-seq data showed BRD4 was associated with *AURKA*; *BUB1B*; *CCNA2*; *CDC25A*; *CENPQ*; *E2F1*,*2*,*5*,*8*; *FOXM1*; *MCM2*,*3*,*5*,*10; USF2*; *SMARCAL1*; and *TOPBP1* in the control HCCLM3 cells, which was reduced upon AZD5153 treatment.

AZD5153 treatment also altered the expression of several polyadenylated lncRNAs and anti-sense RNAs. Among these, expressions of some critical lncRNAs that are known to modulate hepatocarcinogenesis, such as *DANCR* (Yuan et al., 2016), *GATA2-AS1*, *H19* (Matouk et al., 2007), *HOXA-AS2* (Wang et al., 2016), *LINC00152* (Li et al., 2020), *LINC00488* (Gao et al., 2019), *NNT-AS1* (Lu et al., 2017), and *PVT1* (Wang et al., 2014) were suppressed whereas *MALAT1* (Luo et al., 2006), *NEAT1* (Guo et al., 2015), *OSER1-AS1,* and *NORAD* (Yang et al., 2019) were induced in AZD5153 treated HCCLM3 cells (Supplementary Table S4). Collectively, these results suggest that changes in the expression of some of these critical coding and noncoding genes play a causal role in mediating anti-HCC efficacy of this bivalent BRD4 inhibitor.

### AZD5153 and FK866, an NAMPT Inhibitor, Co-Operatively Inhibited HCC Cell Proliferation

**FIGURE 6:**
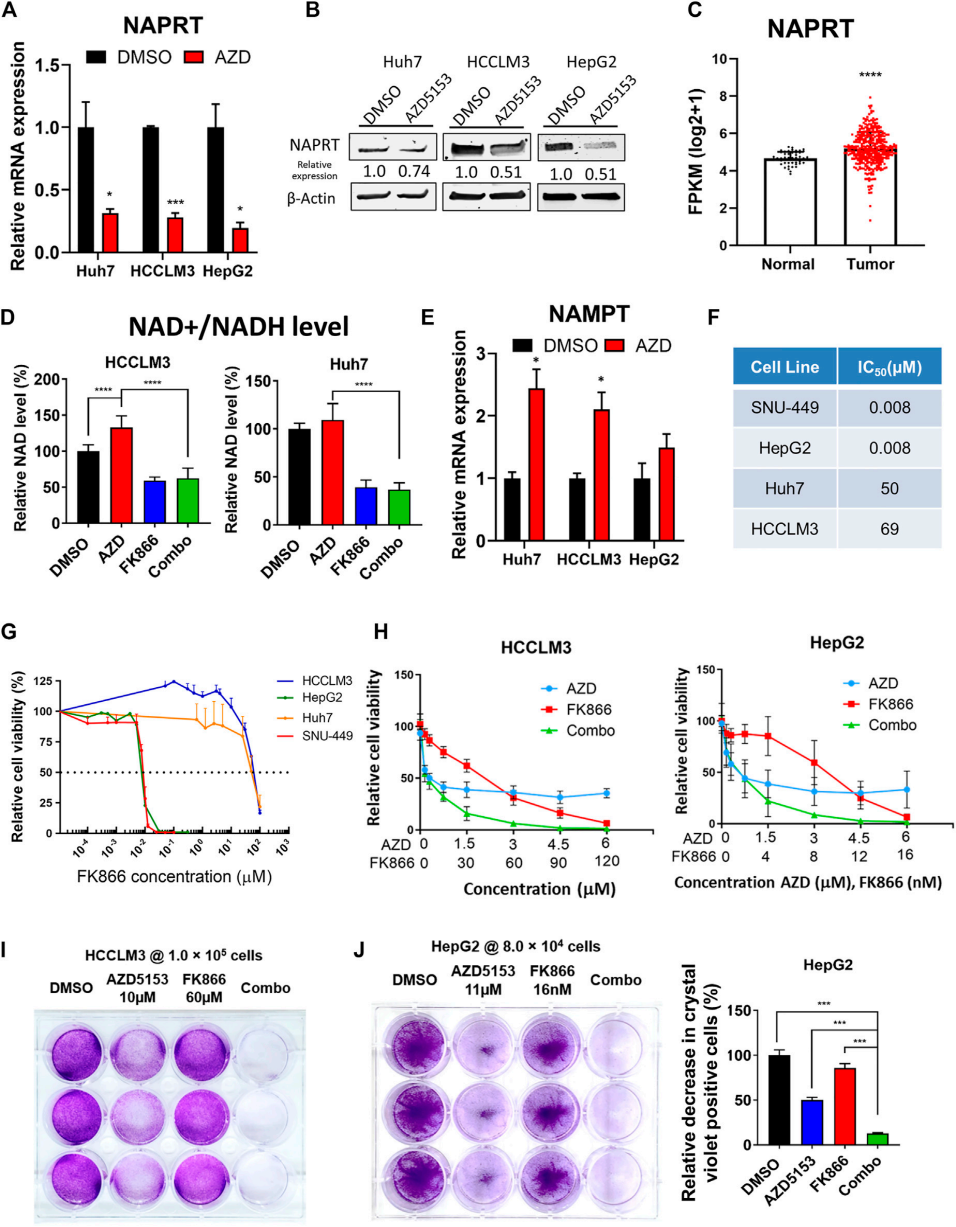
AZD5153 and NAMPT inhibitor (FK866) act in concert to block HCC cell growth. **(A,B)** RT-qPCR **(A)** and immunoblot **(B)** analyses of *NAPRT* in the indicated HCC cell lines treated with AZD5153 (10 μM) for 24 h **(C)** *NAPRT* RNA-seq data in primary HCCs and normal liver tissues were downloaded from LHIC-TCGA database and analyzed using Xena (50 normal and 371 tumor samples). **(D)** Relative levels of total NAD + /NADH levels in the indicated HCC cell lines treated with AZD5153 or FK866 alone and in combination for 72 h. The level in DMSO control was assigned as 100%. Values are mean ± SD (*n* = 3) **(E)** RT-qPCR analysis of *NAMPT* in 3 HCC cell lines treated with AZD5153 (10 μM) for 24 h **(F,G)** IC_50_ of FK866 in HCC cell lines. Cells were treated with different concentrations of the drug and cell viability was measured using CellTiterGlo assay. **(H)** Proliferation of HCC cells treated with different concentrations of AZD5153 or FK866 alone and in combination for 72 h was measured using CellTiter-Glo assay. **(I)** Clonogenic survival assay of HCCLM3 and **(J)** HepG2 cells treated with indicated concentrations of the 2 drugs for 5 days or DMSO and crystal violet positive cells were quantified by colorimetry. *p* values were determined by Student T-TEST. *, **, *** and **** denote *p*-values < 0.05, 0.01, 0.001, and 0.0001, respectively.

We focused on the role of one critical gene RNA-seq data showed that the expression of *NAPRT*, encoding nicotinic acid phosphoribosyltransferase, which catalyzes the first step of NAD^+^ biosynthesis from nicotinic acid, was significantly reduced in AZD5153-treated cells (Figure 5C). This result was confirmed in 3 HCC cell lines treated with AZD5153 by RT-qPCR and Western blot analyses (Figures 6A,B). Furthermore, LIHC-TCGA database analysis revealed significant upregulation of *NAPRT* (Figure 6C) in primary human HCCs compared to normal liver tissues. We, therefore, postulated that AZD5153 might suppress cellular proliferation by lowering NAD^+^/NADH levels through the downregulation of *NAPRT*. Surprisingly, biochemical estimation did not reveal any decrease in these metabolites in HCCLM3 and Huh7 cells; in fact, there was a small but significant increase in their levels in AZD5153-treated cells (Figure 6D). These results led us to measure the expression of *NAMPT,* which encodes nicotinamide phosphoribosyltransferase, the rate-limiting enzyme involved in NAD^+^ synthesis from nicotinamide. Indeed, RT-qPCR data revealed that *NAMPT* was upregulated in the drug-treated HCC cells (Figure 6E). These results led us to posit that AZD5153 and an NAMPT inhibitor may co-operatively inhibit HCC cell proliferation. To test this, we first determined the IC_50_ of FK866, a potent inhibitor of NAMPT, which showed highly variable IC_50_ values among the HCC cell lines tested (Figures 6F,G). SNU-449 and HepG2 cells were most sensitive, whereas HCCLM3 and Huh7 cells were relatively resistant. Next, we treated HCCLM3 and HepG2 cells with different doses of these drugs, alone or in combination, and measured cell viability relative to respective DMSO-treated controls after 72 h. The results showed that both cell lines were relatively resistant to FK866 compared to AZD5153 (Figure 6H). A combination of both drugs, however, was more effective in reducing cell proliferation than treatment with individual drugs at certain concentrations. Clonogenic survival of HCCLM3 and HepG2 cells were also significantly diminished by the drug combination compared to the individual drugs (Figures 6I,J). Assessments of NAD^+^/NADH levels showed that FK866 significantly reduced their levels in both vehicle- and AZD5153-treated HCCLM3 and Huh7 cells (Figure 6D). Collectively, these results suggest that BRD4 and NAMPT inhibitors act in concert by targeting NAPRT and NAMPT, respectively, two key enzymes involved in NAD+/NADH biosynthesis.

## Discussion

In this study, we showed that AZD5153, a bivalent BRD4 inhibitor currently undergoing clinical trials against different solid tumors, inhibits HCC growth both *in vitro* and *in vivo*. Unlike JQ1, a widely used first-line monovalent pan-BRD inhibitor, which requires a relatively high dose (50 mg/kg/day) to treat HCC xenografts in mice (Li et al., 2016), AZD5153 injected IP as lipid nanoemulsions exhibited anti-HCC efficacy at a much lower dose (3 mg/kg/day) in both subcutaneous and orthotopic HCC models without causing systemic toxicity (no significant decrease in body weight compared to the vehicle control). We also elucidated the underlying mechanism of action by identifying genome wide BRD4 binding sites in HCCLM3 cells and their perturbation by the drug, as well as by assessing changes in the gene expression profile of polyadenylated RNAs.

Nanoemulsions are frequently used to deliver hydrophobic drugs to extend drug stability, plasma circulating time, and to enhance bioavailability or solubility (Yukuyama et al., 2017). Here, we encapsulated the hydrophobic BRD4 inhibitor AZD5153 into an MCT-based lipid nanoemulsion to enhance its bioavailability. In the cell-based study, AZD-NE was shown to be less effective compared to the free drug. The shift in IC_50_ of AZD-NE in HCCLM3 cells *in vitro* was mainly caused by the extra barrier in drug release from nanoemulsions. However, formulating AZD5153 into lipid nanoemulsions could extend the plasma circulation time and increase its area under the curve (AUC) in plasma by avoiding renal secretion. An earlier study from AstraZeneca (Rhyasen et al., 2018) indicated that antitumor activity would be enhanced by the long-term target inhibition produced by intravenous infusion of AZD5153, with lipid nanoemulsions serving as a reservoir for sustained release of AZD5153 into the plasma. In a solid tumor model, encapsulating AZD5153 within a lipid nanoemulsion not only enhances the drug circulating time in the plasma but also results in drug accumulation in the tumor site through enhanced permeability and retention (EPR) effect (Fang et al., 2011).

Several studies have shown that BRD4 inhibition is a potential avenue for treating HCC. Multiple mechanisms of HCC suppression by BRD4 inhibition have been posited, including reduction in hyperactivity of enhancers, blockage of expression of fibrogenic proteins, and enhancement of T cell persistence and function (Yang et al., 2020). BRD family members are upregulated in ∼37% of the primary HCC tissues compared with the adjacent non-tumor tissues, which provides a rationale for targeting BRD- mediated pathways for HCC therapy (Li et al., 2016; Choi et al., 2021). Therapeutic efficacy of the first-generation BET inhibitor JQ1 was examined in various HCC cell lines, demonstrating IC_50_ values comparable with those of AZD5153. These authors previously found that JQ1 induced apoptosis in HCC cells by upregulating BIM (Li et al., 2016). Our RNA-seq data also showed that AZD5153 inhibited HCC cell survival by inducing apoptosis that correlated with the induction of BIM. RNA-seq analysis of HCC cells treated with two different BRD4 inhibitors, namely JQ1 or OTX-015, revealed downregulation of genes such as *AOC3*, *CCR6*, *SSTR5*, and *SCL7A11* that are involved in cell migration (Choi et al., 2021).

Research has shown that tumor-bearing mice treated intraperitoneally with JQ1 at a dose of 50 mg/kg/day or 25 mg/kg dosed twice per day exhibited significant tumor growth inhibition and regression in subcutaneous HCCLM3 xenograft models, which correlated with a decrease in Ki-67 positive tumor cells (Li et al., 2016). However, another study showed that a transgenic HCC murine model treated at the same dose and frequency with JQ1 had no significant suppression of tumor growth or tumor numbers, although a trend toward attenuation was noted (Choi et al., 2021). We found that AZD5153 is a potent bivalent selective BRD4 inhibitor of HCC growth both *in vitro* and *in vivo*. Tumor growth inhibition was demonstrated in both subcutaneous and orthotopic HCCLM3 xenograft models at the dose of 3 mg/kg/daily using a lipid nanoemulsion as the carrier. The dose and treatment frequency were much lower than those of JQ1 used in animal studies, indicating that AZD5153 lipid nanoemulsions could be a potential therapeutic candidate in treating HCC.

One report also showed that PD-L1 was upregulated in HCCs upon JQ1 treatment both *in vivo* and *in vitro*, while the combination therapy of JQ1 with anti-PDL1 antibodies in a transgenic HCC model resulted in significant attenuation of tumor growth that correlated with reduction of Ki-67-positive tumor cells in a transgenic HCC model (Liu et al., 2020). Although RNA-seq data did not reveal modulation of PD-L1 expression in HCCLM3 cells by AZD5153, investigation of the efficacy of combination therapies of AZD5153 lipid nanoemulsions with immune checkpoint inhibitors is warranted.

NAD^+^ is a crucial co-factor in diverse biosynthetic and metabolic pathways. Cancer cells reprogram their metabolism to fulfill their need for aggressive proliferation and biomass production. Hence, not only rapid energy production through glycolysis (Warburg effect) but also the synthesis of biomolecular building blocks is critical to tumor progression (Navas and Carnero, 2021). All those processes require NAD^+^ and/or NADP^+^ as co-factors. Interestingly, our study revealed that AZD5153 modulates NAD^+^/NADH metabolism by reciprocally regulating *NAPRT* and *NAMPT* expression in HCC cells *in vitro*. Downregulation of intracellular NAD^+^ level has been shown to induce cellular energy stress and inhibit the mTOR signaling pathway in HCC cells, causing intracellular energy imbalance (Schuster et al., 2015). Combining NAMPT inhibitors with AZD5153, a BRD4 inhibitor, could, therefore, enhance the intracellular energy stress by downregulating key enzymes of the NAD^+^/NADH salvage (NAMPT) and *de novo* (NAPRT) pathways.

## Data Availability

The datasets presented in this study can be found in online repositories. The names of the repository/repositories and accession number(s) can be found in the article/Supplementary Material.
