# Supplementary figures and images for: AZD5153, a Bivalent BRD4 Inhibitor, Suppresses Hepatocarcinogenesis by Altering BRD4 Chromosomal Landscape and Modulating the Transcriptome of HCC Cells
Source: Front Cell Dev Biol. 2022 Mar 24;10:853652. doi: 10.3389/fcell.2022.853652 (PMC8987780; doi:10.3389/fcell.2022.853652)

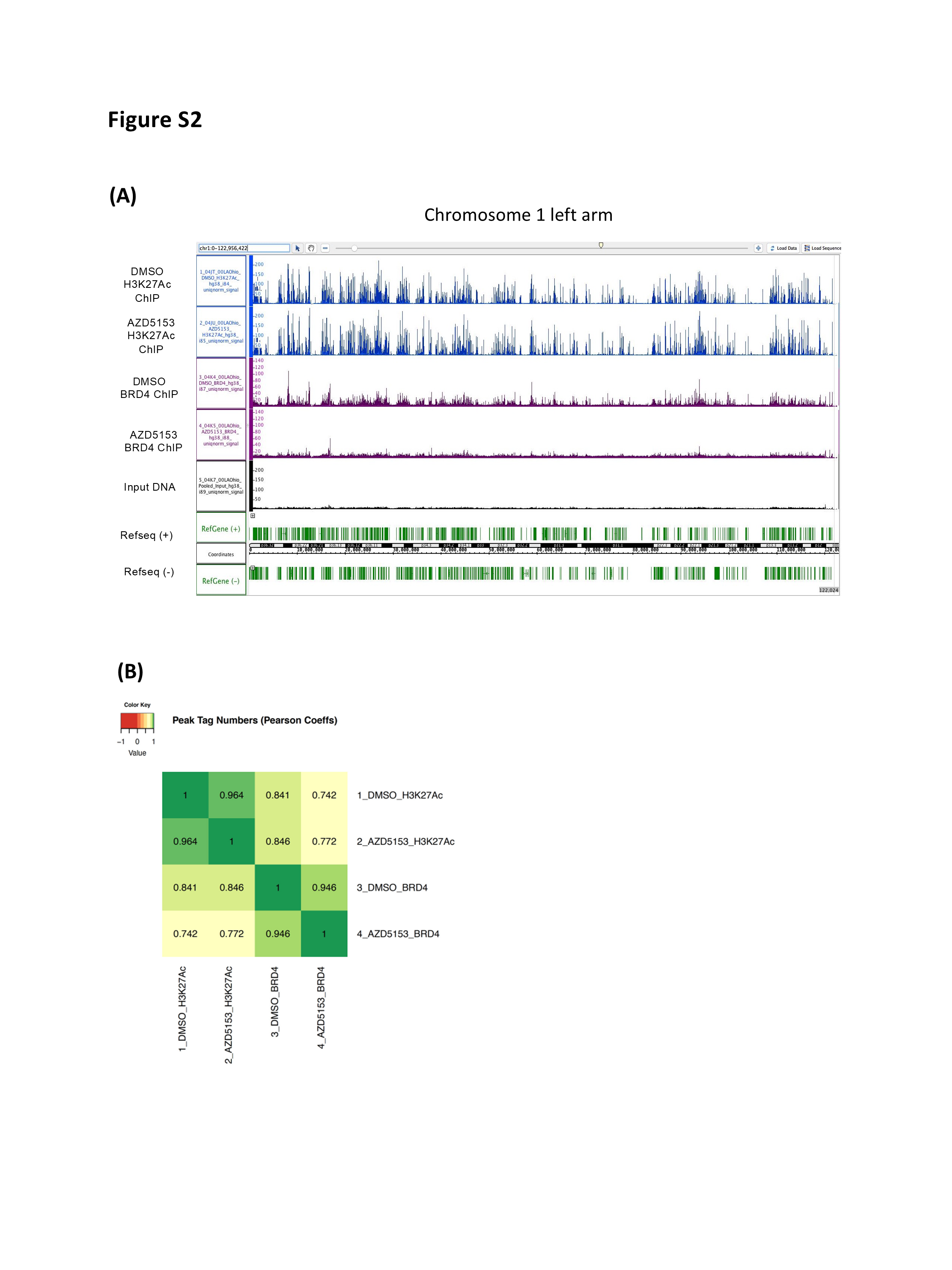

Supplement: Supplementary file 3 [file Image2.TIF]

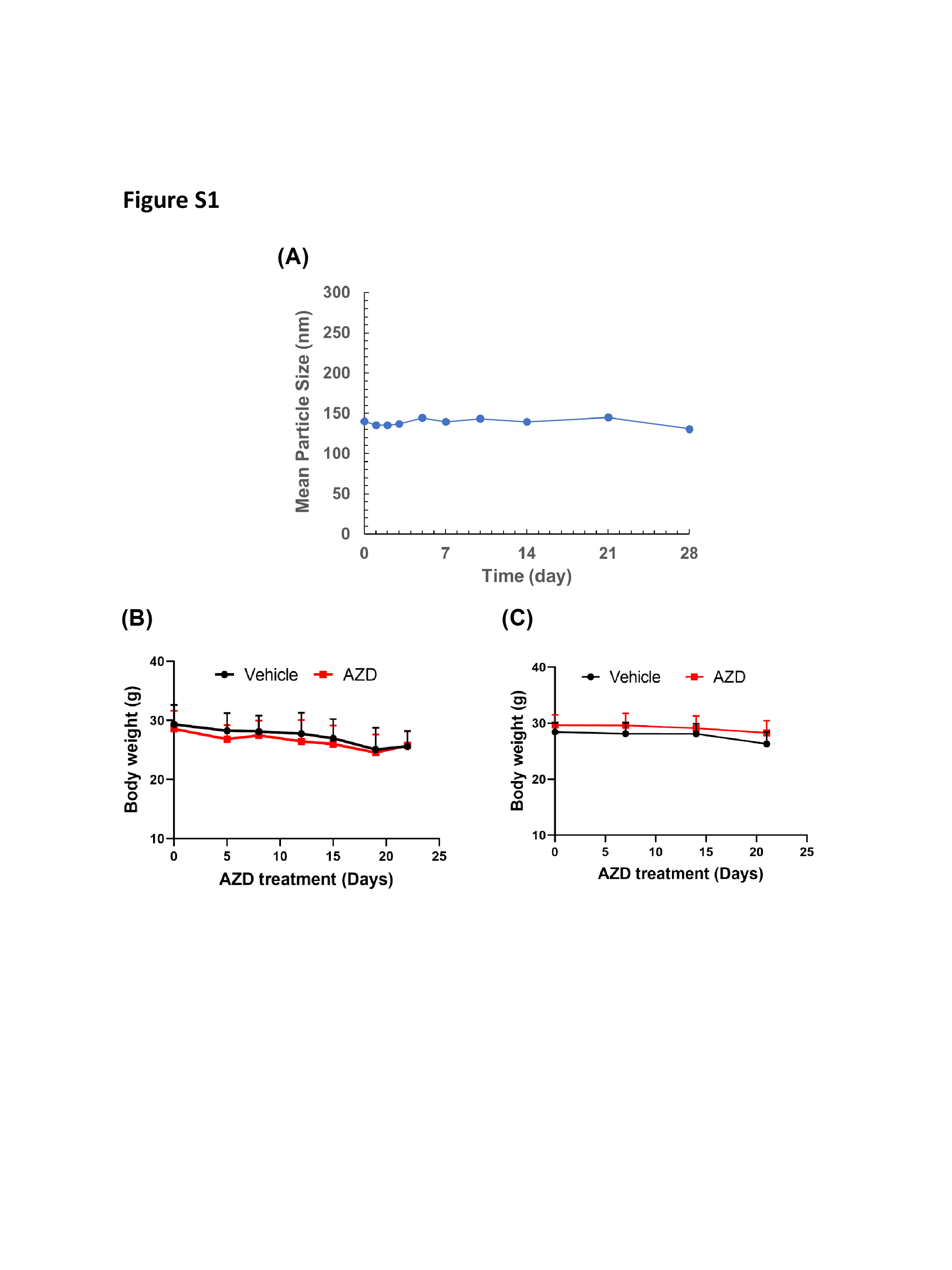

Supplement: Supplementary file 4 [file Image1.TIF]
